# Genomotyping of *Pseudomonas putida* strains using *P. putida* KT2440-based high-density DNA microarrays: implications for transcriptomics studies

**DOI:** 10.1007/s00253-007-0914-z

**Published:** 2007-03-17

**Authors:** Hendrik Ballerstedt, Rita J. M. Volkers, Astrid E. Mars, John E. Hallsworth, Vitor A. Martins dos Santos, Jaçek Puchalka, Joost van Duuren, Gerrit Eggink, Ken N. Timmis, Jan A. M. de Bont, Jan Wery

**Affiliations:** 1 TNO Quality of Life, Business Unit Food and Biotechnology Innovations, Julianalaan 67, 2628 BC Delft, The Netherlands; 2 Wageningen University and Research Center, Agrotechnology and Food Sciences Group, Biobased Products, Bornsesteeg 59, 6708 PD Wageningen, The Netherlands; 3 School of Biological Sciences, MBC, Queen’s University Belfast, BT9 7BL Belfast, Ireland; 4 GBF-National Research Centre for Biotechnology, Mascheroder Weg 1, 38124 Braunschweig, Germany; 5 Faculty of Applied Sciences, Delft University of Technology, P.O. Box 5045, 2600 GA Delft, The Netherlands

**Keywords:** *Pseudomonas putida*, Transcriptomics, Genomotyping, Solvent-tolerant bacteria

## Abstract

*Pseudomonas putida* KT2440 is the only fully sequenced *P. putida* strain. Thus, for transcriptomics and proteomics studies with other *P. putida* strains, the *P. putida* KT2440 genomic database serves as standard reference. The utility of KT2440 whole-genome, high-density oligonucleotide microarrays for transcriptomics studies of other *Pseudomonas* strains was investigated. To this end, microarray hybridizations were performed with genomic DNAs of subcultures of *P. putida* KT2440 (DSM6125), the type strain (DSM291^T^), plasmid pWW0-containing KT2440-derivative strain mt-2 (DSM3931), the solvent-tolerant *P. putida* S12, and several other *Pseudomonas* strains. Depending on the strain tested, 22 to 99% of all genetic elements were identified in the genomic DNAs. The efficacy of these microarrays to study cellular function was determined for all strains included in the study. The vast majority of DSM6125 genes encoding proteins of primary metabolism and genes involved in the catabolism of aromatic compounds were identified in the genomic DNA of strain S12: a prerequisite for reliable transcriptomics analyses. The genomotypic comparisons between *Pseudomonas* strains were used to construct highly discriminative phylogenetic relationships. DSM6125 and DSM3931 were indistinguishable and clustered together with strain S12 in a separate group, distinct from DSM291^T^. *Pseudomonas monteilii* (DSM14164) clustered well with *P. putida* strains.

## Introduction

The sequencing and annotation of the *Pseudomonas putida* KT2440 genome (Nelson et al. 2002) has greatly catalyzed research on this strain and other academically and biotechnologically relevant but non-sequenced *P. putida* strains. A number of key scientific advances (both pure and applied) have been made via proteome and transcriptome analysis of *P. putida* strains (Dominguez-Cuevas et al. 2006; Hallsworth et al. 2003; Morales et al. 2006; Reva et al. 2006; Santos et al. 2004; Segura et al. 2005; Volkers et al. 2006; Yuste et al. 2006). In our laboratories, we have studied both *P. putida* KT2440 (for recent publications: Hallsworth et al. 2003; Martins dos Santos et al. 2004; Nelson et al. 2002; Timmis 2002) and the solvent-tolerant *P. putida* S12 (De Bont 1998; Hartmans et al. 1990; Wery and De Bont 2004). Unlike most pseudomonads, solvent-tolerant strains possess an extraordinary tolerance to a broad variety of toxic solvents (for reviews: De Bont 1998; Kieboom and De Bont 2000; Ramos et al. 2002). A large number of high-added value bioconversions involve toxic, generally apolar, products (aromatics, aliphatic alcohols, epoxides, etc.). Especially for bioprocesses involving such products, the use of solvent-tolerant *P. putida* strains renders advantages in terms of productivity and the application of multiphase media for product recovery (Ramos-Gonzalez et al. 2003; Rojas et al. 2004; Wery and De Bont 2004; Wery et al. 2000; Wierckx et al. 2005). Despite their biotechnological potential, the genomes of S12 and other *Pseudomonas* strains have not been sequenced. So, recent studies of their metabolic potential have been limited to comparative proteomics (Segura et al. 2005; Volkers et al. 2006) or transcriptomics based on the database information for *P. putida* KT2440.

The genus *Pseudomonas* is very heterogeneous (Anzai et al. 2000); even strains of one species tend to vary greatly in terms of both phenotypic (Grimont et al. 1996) and genotypic characteristics (Brosch et al. 1996). Strains belonging to the species *P. putida* can be categorized into biovar A and B: the former grouping (A) is the more heterogeneous (Brosch et al. 1996) and the phylogenetic and metabolic diversity of biovar A strains has yet to be fully characterized and industrially exploited.

Comparative transcriptomics-based approaches have played a pivotal role in recent investigations of complex cellular responses of *P. putida* strains (Dominguez-Cuevas et al. 2006; Duque et al. 2007; Yuste et al. 2006). As transcriptome profiling is based on the highly-sensitive detection of DNA–cDNA hybridization, DNA sequence similarity determines the validity of these analyses. The inherent heterogeneity of the *P. putida* grouping provides additional cause for concern that microarray platforms derived from strain KT2440 may provide a valid framework for the study of nonsequenced *P. putida* strains.

We therefore carried out this study to assess the utility of KT2440-based high-density DNA microarrays for transcriptomics studies of DSM 6125 (subculture of KT2440), DSM 3931 (subculture of mt-2), DSM 291^T^ (the *P. putida* type strain), the solvent-tolerant S12, and other nonsequenced *Pseudomonas* strains. In addition, the use of such microarrays to establish highly discriminative phylogenetic relationships between these strains was demonstrated.

## Materials and methods

### Strains, cultivation conditions, and DNA extraction

Single colonies from each *Pseudomonas* strain studied (see Table 1) grown on Pseudomonas Isolation Agar (Difco) were used to inoculate Luria–Bertani broth (LB) (Sambrook et al. 1982) in duplicate. After cultures were grown overnight at 30°C, genomic DNAs were prepared from 5 ml of culture (approximately 3 × 10^8^ cells/ml) using the Genomic DNA 100/G Kit (Qiagen, Germany) according to the manufacturer’s instructions. DNA concentrations were determined at 260 nm using ND-1000 spectrophotometer (NanoDrop, Wilmington, DE, USA), and purity was confirmed by agarose (1% *w*/*v*) gel electrophoresis.

**Table 1:** Source and designations of *Pseudomonas* strains used in this study

Species and strain number^a^	Source or reference^b^	Other designations^c^
*P. putida* DSM 6125	DSMZ	KT2440, ATCC 47054; NCIMB 11950
*P. putida* DSM 3931	DSMZ	mt-2, ATCC 23973; ATCC 33015; JCM 6156; NCIMB 12182
*P. putida* S12	Hartmans et al. 1990	ATCC 700801
*P. putida* DSM 291^T^	DSMZ	DSM 50202^T^; ATCC 12633^T^; ICPB 2963^T^; NCTC 10936^T^; CCUG 12690^T^; LMG 2257^T^
*P. putida* DSM 50198	DSMZ	ATCC 17453; ICPB 2563-77; JCM 6157; NCIMB 10007
*P. putida* DSM 50208	DSMZ	ATCC 17485; ICPB 2789-111; JCM 6158; NCIMB 12092
*P. monteilii* DSM 14164	DSMZ	ATCC 700476; CCUG 38736; LMG 21609; CIP 104883
*P. fluorescens* DSM 50090^T^	DSMZ	ATCC 13525^T^; ICPB 3200^T^; NCIMB 9046^T^; NCTC 10038^T^; CCUG 1253^T^; LMG 1794^T^

^a^Strain designations used in this study

^b^*DSMZ* Deutsche Sammlung von Mikroorganismen und Zellkulturen, Germany

^c^Subcultures of the used strains available in other culture collections. *ATCC* American Type Culture Collection, *ICPB* International Collection of Phytopathogenic Bacteria, USA, *NCIMB* National Collections of Industrial and Marine Bacteria, UK, *NCTC* National Collection of Type Cultures, UK, *JCM* Japan Collection of Microorganisms, Japan, *CIP* Collection bacteriènne de l’Institut Pasteur, France, *CCUG* Culture Collection University Göteborg, Sweden, *LMG* BCCM/LMG Bacteria Collection, Belgium.

### Preparation of biotin-labelled fragmented genomic DNAs

Approximately 17 μg of heat-denatured genomic DNA was fragmented using 0.3 U DNaseI (Pharmacia)/μg DNA at 37°C for 20 min (Wolfgang et al. 2003). DNaseI was inactivated by immediate heat treatment at 95°C for 10 min, followed by cooling on ice. An aliquot of fragmented DNA was analyzed by agarose (4% *w* / *v* in TAE buffer) gel electrophoresis to confirm the purity and establish the mean size of DNA fragments (20–50 bp). Approximately 7 μg of fragmented DNA was labelled with biotin according to the Affymetrix “Expression Analysis Technical Manual for Prokaryotic Samples”.

### Design of high-density oligonucleotide microarrays and hybridization with genomic DNAs

High-density oligonucleotide microarrays based on the annotated genome of *P. putida* KT2440 (NC 002947.3) were constructed according to Affymetrix specifications (http://www.affymetrix.com/support/technical/other/custom_designmanual.pdf ) with the pair-wise configuration of 13 perfect match (PM) and mismatch (MM) 25-mer oligonucleotides per gene (probe set). The microarrays included 7,781 probe sets: 5,338 representing 5,350 annotated genes or open reading frames (orfs), and 2,443 targeting intergenic regions. Hybridization and washing of the microarrays were performed on a GeneChip® Node system (Hybridization temperature 49°C; Hybridization oven 640, Fluidics station 450; Affymetrix, Santa Clara, CA, USA) following the supplier’s instructions. Scanning was carried out by ServiceXS (Leiden, The Netherlands) on a high resolution Gene Chip® Scanner 3000 7G system with autoloader (Affymetrix, Santa Clara, CA, USA) using the default analysis settings of the manufacturer (filter: 570 nm; pixel size: 2.5 μm). Hybridization intensity data were extracted from the scanned array images, and the designations for presence or absence were calculated with Affymetrix Microarray Suite (MAS) 5.0 software. A one-sided Wilcoxon’s Signed Rank was used to calculate *p*-values reflecting the statistical significance of differences between PM and MM of a probe set. The significance levels for detection calls of probe sets were *p*-value < 0.05 for present and *p*-value ≥ 0.05 for absent. Differences between PM and MM were considered insignificant and removed from further consideration by comparisons of the discrimination score [(PM−MM)/(PM+MM)] with the defined discrimination threshold *τ* (discrimination score<0.015). Comparisons of present/absent designations for genes in the different strains were made with GeneSpring version 7.2 (Agilent). Replicate assays of biological duplicates were performed for *P. putida* DSM 6125 and S12. The deviation caused by present/absent designations of genes in only one of both replicates was 0.8% for DSM 6,125 and 4.2% for S12 regarding all probe sets for putative genes and intergenic regions (Table 2).

**Table 2:** Similarity indices based on designations of presence or absence (stated in brackets) from hybridization signals of genomic DNA fragments from different *Pseudomonas* members on *P. putida* KT2440-based microarrays^a^

Strain	Present (absent) in percent (%)		
	Putative genes and intergenic regions (7,781 probe sets)	Putative genes (5,338 probe sets)	Putative genes with assigned function (3,670 probe sets)
*P. putida* DSM 6125^b^	97.6 (1.4)	99.6 (0.0)	99.8 (0.0)
*P. putida* DSM 3931	98.6 (1.4)	99.9 (0.1)	100.0 (0.0)
*P. putida* S12^b^	78.0 (16.9)	81.8 (13.9)	86.9 (9.7)
*P. putida* DSM 291^T^	60.5 (39.5)	64.2 (35.8)	69.3 (30.7)
*P. putida* DSM 50198	56.2 (43.8)	60.7 (39.3)	66.5 (33.5)
*P. putida* DSM 50208	58.8 (41.2)	62.6 (37.4)	67.8 (32.2)
*P. monteilii* DSM 14164	57.7 (42.3)	61.7 (38.3)	66.8 (33.2)
*P. fluorescens* DSM 50090^T^	22.0 (78.0)	27.2 (72.8)	30.7 (69.3)

^a^Present/absent designations (present: *p* < 0.05; absent: *p* ≥ 0.05; *τ* = 0.015) derived from a decision matrix in Affymetrix MAS 5.0

^b^Replicate assays were performed for *P. putida* DSM 6125 and S12 and the values listed include only genes that were designated present or absent in both replicates.

### Calculation of dendrograms

Simple matching similarity matrix based on the present/absent detection calls in microarray analysis of different pseudomonads (SM = *m*/*n*; *m*, number of matching probe sets; *n*, total number probe sets), and the microarray genotyping dendrogram were calculated using UPGMA parameters (unweighted pair group method, arithmetic average) and agglomerative hierarchical clustering with XLSTAT version 7.5.3 (Addinsoft, Paris, France).

### AFLP analysis

In this paper, we used amplified fragment length polymorphism (AFLP) (Janssen et al. 1996; Savelkoul et al. 1999; Vos et al. 1995) to analyze different strains of *P. putida* a *P. monteilii* and a *Pseudomonas fluorescens*. AFLP is based on selective amplification of restriction fragments from totally digested genomic DNA. AFLP fingerprints were performed by KeyGene (Wageningen, The Netherlands). Genomic DNA of the different pseudomonads was digested using restriction enzyme combination *Nla*III (Westburg, Leusden, The Netherlands) and *Csp*6I (Fermentas, St. Leon-Rot, Germany) according to manufacturers instructions. Each restriction enzyme was combined with the ligation of specific linker oligonucleotide pairs (*Nla*III: 5-GACGATGAGTCCTGAG-3/5-TGTACGCAGTCTAC-3; *Csp*6I: 5-GACGAT GAGTCCTGAG-3/5-TACTCAGGACTCAT-3). For each of these linker combinations, AFLP was performed using nine N/C AFLP primer combinations, which were selected using KT2440 genome sequence as reference and software package REcomb (Keygene) for prediction analysis. These primers were extended with a 3′ terminal dinucleotide (+ 2) and the extensions were CA/AC, CA/CA, CA/CC, CA/GG, CA/TT, CT/TT, CT/CA, CT/CT, and CT/TC. For further detail, please refer to Van den Braak et al. (2004). PCRs were performed in the presence of radioactive nucleotides, and the amplimers obtained were separated on 50 × 20 cm polyacrylamide slabgels. Using phosphor-imaging, the individual presence/absence in a total of 757 markers (DNA bands) per strain was analyzed.

The total marker score data table (presence/absence of individual DNA bands) was subjected to genetic distance analysis using simple matching similarity matrix (SM = *m*/*n*; *m*, the number of matched scores; *n* the total sample size), consisting of similarity indices for all combinations of AFLP-banding patterns. Simple matching coefficients were calculated using NTSYSpc-software version 2.2 (Exeter Software, Setauket, NY, USA). To visualize the relationship between the strains, a dendrogram was generated using Sequential Agglomerative Hierarchical Nested (SAHN) cluster analysis with the use of UPGMA parameters.

### Standard PCR and sequencing

Diagnostic PCR of selected putative orfs (PP1265, PP5224) that were called absent in microarray analyses of *P. putida* DSM 3931 genomic DNA was performed using proofreading enzyme mixture High Fidelity PCR master (Roche Diagnostics, The Netherlands) and specific primers (PP1265: forward: 5′-CTGCTGCACCAGGCCTAT-3′, reversed: 5′-TTGGTCACATAGCCGTCAAC-3′; PP5224: forward: 5′-CAACGGCTAAACCTTTGCAT -3′, reversed: 5′-AGGATCGAGACCTTGCCTTC-3′). Yielded PCR-amplicons of expected sizes, 1,108 and 1,062 bp, respectively, were sent to Baseclear (Leiden, The Netherlands) for sequencing according to Sanger et al. (1977) using nested sequencing primers (PP1265: 5′-CCAGGCAATCCGTGTCAT-3′; PP5224: 5′-GGTGTCCTGACCGTCAAGTT-3′), and the resulting sequences were used for sequence alignments.

### Biological function-derived phylogenetic analysis

The concept of Clusters of Orthologous Genes (COG; see Tatusov et al. 1997, 2003) was used during analysis of the genomic content of nonsequenced *Pseudomonas* strains by linkage to primary biological function (Table 3). A COG consists of individual proteins or groups of paralogs from at least three lineages and thus corresponds to an ancient conserved domain (Tatusov et al. 1997, 2003). In the NCBI-COG database, 4,497 proteins of 5,350 putative orfs in *P. putida* KT2440 were identified as COGs and were arranged in functional categories (see http://www.ncbi.nlm.nih.gov/sutils/coxik.cgi?gi=266 ). Because of the limited number of COGs in the functional categories (A) RNA processing and modification, (B) chromatin structure/dynamics, and (W) extracellular structures (1, 2, and 3, respectively), these COGs were collectively grouped together with general function prediction COGs (Category R) under the designation R′(see Table 3).

**Table 3:** Primary functional designation^a^ of genes identified in *P. putida* S12 genomic DNA

Code^b^	Description	COGs in KT2440^c^	COGs KT2440 in S12
F	Nucleotide transport and metabolism	93	87 (93.5%)
J	Translation	194	178 (91.8%)
H	Coenzyme transport and metabolism	183	166 (90.7%)
T	Signal transduction mechanisms	427	387 (90.6%)
I	Lipid transport and metabolism	194	174 (89.7%)
N	Cell motility	130	116 (89.2%)
G	Carbohydrate transport and metabolism	264	232 (87.9%)
P	Inorganic ion transport and metabolism	368	322 (87.5%)
U	Intracellular trafficking and secretion	119	104 (87.4%)
E	Amino acid transport and metabolism	630	550 (87.3%)
O	Posttranslational modification, protein turnover, chaperones	194	169 (87.1%)
C	Energy production and conversion	321	276 (86.0%)
Q	Secondary metabolites biosynthesis, transport and catabolism	161	138 (85.7%)
K	Transcription	499	426 (85.4%)
V	Defense mechanisms	66	56 (84.8%)
R′	General function prediction only	733	620 (84.6%)
S	Function unknown	442	365 (82.6%)
M	Cell membrane biogenesis	288	227 (78.8%)
D	Cell cycle control	56	41 (73.2%)
–	Not in COGs	853	544 (63.8%)
L	Replication, recombination and repair	269	158 (58.7%)

^a^Based on the Clusters of Orthologous Groups (COG) system, also see Materials and methods section)

^b^Codes of functional categories of COG

^c^Proteins, 4,497 of 5,350 putative orfs in *P. putida* KT2440 can be found in COG database (http://www.ncbi.nlm.nih.gov/COG/)

## Results

### Genomic DNA hybridizations with *P. putida* KT2440-based high-density DNA microarrays

Total genomic DNA from *P. putida* strains, and other nonsequenced *Pseudomonas* members (see Table 1), was hybridized to custom KT2440-based high-density oligonucleotide microarrays. Presence or absence designations for each probe set (designed for specific genes and intergenic regions) were calculated by the Affymetrix MAS 5.0 algorithm from the significant difference (see Materials and methods section) in hybridization intensities between the corresponding perfect match and mismatch oligonucleotides (Table 2). Absence designation is a synonym for the absence of significant signal values and stands for divergent DNA still coding a similar biological function or for the complete absence of the specific DNA. Replicate array hybridizations were performed for DSM 6125 DNA and S12 DNA only. For these DNAs the values given in Table 2 represent only probe sets designated present or absent in both replicates. As expected, the DSM 6125 DNA yielded an almost perfect score: 97.6% for probe sets corresponding to all genetic elements (including the intergenic regions) and 99.8% for probe sets designed for genes with an assigned function. Strain DSM 3931 (subculture of *P. putida* mt-2: Teruko, 2002) is a TOL plasmid (pWW0)-containing variant of DSM 6125 and was used as an additional control for the accuracy of the microarray experiments. As expected, both strains were virtually indistinguishable in the array hybridization study (Table 2). Nonetheless four orfs were indicated absent in DSM 3931. These orfs were found to be called absent in only one of the DSM 6125 replicates. We therefore used diagnostic PCR to investigate the presence of two of them (PP1265, PP5224) in DSM 3931; the other two appeared less important due to their limited size (~90 bp). In both cases, PCR products of the expected sizes (1,108 and 1,062 bp) were obtained that, after sequencing, proved to be identical to the KT2440 homologs.

Apart from DSM 3931, the solvent-tolerant S12 showed the highest genomic similarity to KT2440. Nearly 3,188 of 3,670 (86.9%) genes with assigned functions in the KT2440 genome were identified in the genomic DNA of S12 (Table 2). Approximately 71% of 1,668 (putative) genes without an assigned function, and 70% of the intergenic regions were found to be present in the S12 (data not shown).

### Microarray-based genomotyping

**Fig. 1:**
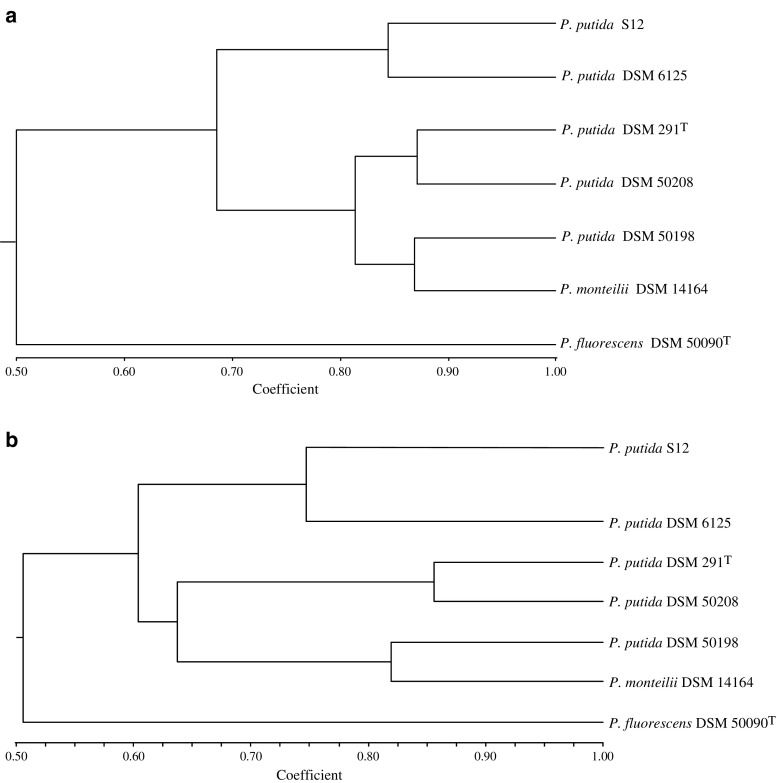
Genome similarity between different *Pseudomonas* strains. **a** High-density microarray genotyping tree based on absent/present designations generated by Affymetrix MAS 5.0 algorithm (Table 2) using simple matching similarity coefficient, UPGMA, and agglomerative hierarchical clustering. **b** AFLP-genotyping. Tree is based on the score of 757 AFLP markers using the simple matching similarity coefficient and Sahn cluster analysis

A dendrogram was constructed (Fig. 1a) based on genomic similarity of all 7,781 genetic elements of KT2440 in the tested genomic DNAs except for the control strain DSM 3931 (Table 2). DSM 6125, DSM 3931 (not shown), and S12 clustered in a group separate from the other *P. putida* strains (Fig. 1a). The nonsequenced *P. fluorescens* (DSM 50090^T^), that was included as an out-group, did not cluster with any other strain. By contrast, *Pseudomonas monteilii* DSM 3931 that was included as a non-*putida* member clustered with other *P. putida* strains suggesting a closer relationship with the *P. putida* taxonomic grouping (Fig. 1). To assess the validity of these microarray-derived phylogenetic relationships, AFLP DNA fingerprinting was used to obtain an independent phylogenetic classification of strains (Janssen et al. 1996; Savelkoul et al. 1999; Vos et al. 1995). The phylogenetic tree constructed following AFLP analyses showed an identical pattern in terms of strain grouping. (Fig. 1b).

**Fig. 2:**
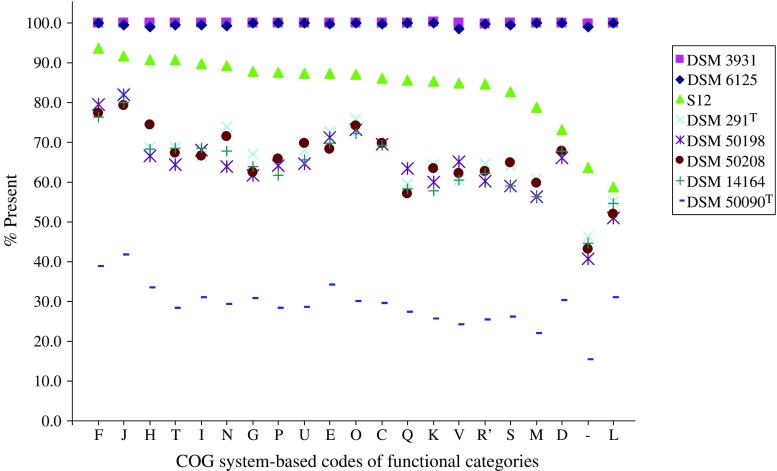
Distribution of genes encoding COG proteins over different functional categories as identified in *Pseudomonas* strains. Functional categories were adopted from the COG database for *P. putida* KT2440 (http://www.ncbi.nlm.nih.gov/sutils/coxik.cgi?gi=266 ). The categories of biological function corresponding to COG codes are given in Table 3.

An inventory was made of genes encoding proteins belonging to COGs (Tatusov et al. 1997, 2003) that were identified in the different genomic DNAs (Fig. 2). Of 5,350 orfs in the KT2440 genomic DNA sequence, 4,497 encode proteins that have been categorized into classes of primary biological function based on the COG system (http://www.ncbi.nlm.nih.gov/COG/). It was found that, after DSM 3931, *P. putida* S12 showed the highest present score in all functional classes (Fig. 2). The “present” designations for S12 genes encoding COG members ranged from 58.7 to 93.5%, depending on their primary biological function (Table 3, Fig. 2). The unequal distribution of the present designations over the different functional classes was also characteristic of other *Pseudomonas* strains (Fig. 2). Strikingly, all pseudomonads other than DSM 6125, DSM 3931, S12 and the out-group DSM50090^T^ showed a comparable distribution pattern of identified genes over the different COGs.

*P. putida* strains are well-known for their broad metabolic potential regarding aromatic compounds (Jimenez et al. 2002; Wackett 2003, for reviews). Several pseudomonads, including *P. putida* KT2440, have been reported to degrade and/or transform a variety of aromatics. Among these are compounds of industrial importance, and there is an interest in studying these metabolic pathways on the level of gene expression and regulation thereof. The utility of KT2440-based microarrays in transcriptomics studies of aromatics metabolism of the pseudomonads under investigation was appraised. The presence of 70 genes of central and peripheral metabolic routes of aromatics were chosen based on reports by Jimenez et al. (2002) and Wackett (2003) (Table 4). All genes (100%) were detected in DSM 6125, S12 and in DSM 3931. Higher divergence was found for *P. putida* DSM 291^T^ (82.9% detectable), DSM 50198 and DSM 50208 (both 75.7% detectable), and DSM 14164 (74.3% detectable). DSM 50090^T^ again behaved as an out-group with only 25.7% of the 70 genes detectable. In the *P. putida* strains used in the present study and DSM 14164, almost all genes for degradation of benzoate (*ben*), homogentisate (*hmg, mai*), phenylalanine/tyrosine (*phh, tyrB*), and *catAB* were detected. In DSM 291, 50198, 50208 and 14164 putative regulatory genes of different pathways (e.g. *catR, pcaQ, pobR, phaNM*) and some isoenzymes (e.g., *catA2*) were not found (Table 4). Interestingly, in all these strains, the muconolactone isomerase (*catC*), and in the catabolism of phenylpropenoid compounds, vanillin dehydrogenase (*vdh*), putative conifer aldehyde dehydrogenase (*calB*; except DSM 50198), and feruloyl-CoA synthetase (*fcs*; except for DSM 50208) were not identified and can thus not be studied in KT2440-based microarray-based transcriptome analysis.

**Table 4:** Comparison of the presence/absence designations of genes encoding metabolic pathways of aromatic compounds in different pseudomonads derived from Affymetrix decision matrix MAS 5.0

Strain	Present (%)^a^	Genes of aromatic pathways not identified in hybridizations with genomic DNA
*P. putida* DSM 6125	100.0	
*P. putida* DSM 3931	100.0	
*P. putida* S12	100.0	
*P. putida* DSM 291^T^	82.9	*catC; fadAxB2x; fcs; pcaQ; phaDHMN; vdh; calB; catA2*
*P. putida* DSM 50198	75.7	*benE-1; catCR; ech; fadAxB2x; fcs; pcaDQ; phaGHMN; pobAR; vdh; catA2*
*P. putida* DSM 50208	75.7	*catCR; ech; fadAxB2x; pcaBCQ; phaDHJMN; phhR; pobR; vdh; calB; catA2*
*P. monteilii* DSM 14164	74.3	*benE-1; catCR; ech; fadB2x; fcs; pcaBDQ; phaEGHMN; pobAR; vdh; calB; catA2*
*P. fluorescens* DSM 50090^T^	25.7	*aat; acdA; benACDE-1E-2FKR; catABCR; fadAxBB1xB2xDx; fcs; hpd; pcaCDHIJKQT; pcm; phaAB-EG-ILMN; phhBR; pobAR; tyrB-2; vanAB; vdh; calB; ferR; catA2*

^a^Selected 70 genes (100%) involved in catabolism of aromatic compounds annotated in *P. putida* DSM 6125: protocatechuate (*pcaBCDFGHIJKQRT*), catechol branches β-ketoadipate pathway (*catABCRA2*), homogentisate pathway (*hmgA, mai*), central *meta*-cleavage (*pcm*), p-hydroxybenzoate (*pobAR*), benzoate (*benABCDE-1E-2FKR*), phenylacetate pathway (*phaABCDEFGHIJKLMNJ1*), n-phenylalkanoic acids (*fadABDHAxB1xB2xD2Dx*), phenylpropenoid compounds (*vanAB, vdh, calB, ferR, fcs, ech, aat, acdA*), and phenylalanine/tyrosine (*phhABR, tyrB-1B-2, hpd, pcm*).

## Discussion

A major advantage of microarray-based comparisons of species is the ability to pinpoint differences in individual genes and intergenic regions. Through these comparisons, detailed insight was gained in the utility of *P. putida* KT2440-based microarrays in transcriptomics studies of different pseudomonads at the level of specific categories of biological function. It was found that genes involved in, e.g., “translation” and “nucleotide transport and metabolism” could be identified at a high frequency (>80%) in all *P. putida* strains tested, in contrast to other functional groups where the frequency of gene identification dropped below 60%. Whereas *P. fluorescens* DSM 50090^T^ behaved as a typical out-group in these functional studies, it was clear that, depending on the functional category, up to 45% of the genes of this strain could still be identified.

There is a biotechnological requirement for *P. putida* biocatalysts that can function at high solvent concentrations, such as strain S12, and that can be swiftly optimized for different bio-based production processes. In our group, studies have focused on the construction of strains that are able to convert renewable substrates, such as sugars, into aromatics of interest via central metabolites (Nijkamp et al. 2005, 2006; Wierckx et al. 2005). These conversions take place via multiple metabolic pathways each consisting of several enzymatic steps with regulatory mechanisms that are being investigated using S12 as a model system. A comparative transcriptomics approach is invaluable to gain detailed insights into the complex cellular systems of the metabolically versatile pseudomonads. The employment of the high-density KT2440 microarrays would enable highly sensitive and reproducible transcriptome analyses that are compatible with those used for model species such as *P. aeruginosa* (Ochsner et al. 2002; Wagner et al. 2003; Whiteley et al. 2001) and *Escherichia coli* (Woo et al. 2004).

In the present study, we showed that the use of KT2440-based microarrays would enable reliable transcriptomics analysis of *P. putida* S12. Significantly, we found that of all pseudomonads tested, the genomic content of *P. putida* S12 showed the highest similarity to that of *P. putida* DSM 6125 (KT2440). The vast majority of KT2440 genes coding for proteins involved in primary metabolism, including biosynthesis of important intermediates such as amino acids, and the genes involved in the conversion of aromatic compounds were shown to be sufficiently similar to those of S12.

The high resolution achieved by comparative genomotyping enabled the identification of minute genotypic differences between tested strains, making a meaningful analysis of phylogenetic relationships feasible. For example, the genomic DNA of *P. putida* DSM 6125 was shown to be virtually identical to that of the control strain DSM 3931, and this is consistent with the origin of strain KT2440 as a derivative of strain mt-2 (Regenhardt et al. 2002).

The relationship between the *P. putida* DSM 291^T^ and KT2440 has been an issue of controversy. Based on 16S rRNA gene comparisons (99% identity), both strains appeared closely related; however, a DNA–DNA hybridization experiment indicated only 50.5% genome relatedness between both strains (Regenhardt et al. 2002). The results lead to doubts about the classification of both strains as part of the same *Pseudomonas* species, given the widely accepted recommendation that strains of the same species shall have genome similarities higher than 70% (Stackebrandt and Goebel 1994). In the same study, an appreciable distance between DSM 291^T^ and KT2440 was established by REP-PCR genomic fingerprint patterns and Biolog GN metabolic profiling. In our genomotyping approach, 69.3% of the genes with an assigned function, 60.5% of all genetic elements (including intergenic regions), and 52.2% of the intergenic regions (not shown) were identified in the genomic DNA of *P. putida* DSM 291^T^. These differences, which are supported by the AFLP analysis, indicate a considerable phylogenetic distance between DSM 291^T^ and KT2440.

The diversity within the species *P. putida* was previously reflected in studies concerning genomic DNA ribotyping (Brosch et al. 1996), whole cell protein electrophoretic fingerprinting (Vacanneyt et al. 1996) and Biolog/Biotype-100 experiments (Grimont et al. 1996). In our genomotyping studies, strains of *P. monteilii* and *P. fluorescens* were included as out-groups. *P. fluorescens* DSM 50090^T^ was shown to be distantly related to the other *Pseudomonas* members tested, which supports its classification as a separate species. However, *P. monteilii* DSM 14164 clustered well with *P. putida* DSM 50198 and to a lesser extent with DSM 291^T^ and *P. putida* DSM 50208. AFLP analysis showed a comparable result and confirmed the close relation between *P. putida* and *P. monteilii*. The present study thus suggests that DSM 14164 should more accurately be classified as a *P. putida*.

Other studies based on classification of *P. monteilii* by classical, well established taxonomic methods do not support our findings. DNA–DNA hybridizations among *P. monteilii* CFML 90-60^T^, DSM 291^T^, and DSM 50208 generated relative bindings of genomic DNA of 40 and 48%, with Δ*T*_m_ values of 9.2 and 7.9, respectively (Elomari et al. 1997). Pyoverdine typing (siderotyping) analyzing the excreted siderophores during iron starvation of *P. monteilii* CFML 90-60^T^ and DSM 291^T^ produced different patterns for both strains (Dabboussi et al. 2002). Phenotypically, however, *P. monteilii* was previously shown to be highly similar to *P. putida* and could only be differentiated by assimilation experiments of the substrates inositol, α-aminobutyrate, and *o*-/*m*-hydroxybenzoate (Dabboussi et al. 2002; Elomari et al. 1997).

In conclusion, the genomotyping of different pseudomonads using KT2440-based DNA microarrays yielded novel insights in their phylogenetic relationships and the underlying identification of genes and their distribution over different primary and secondary biological functions. This revealed the utility of KT2440-based microarrays in transcriptomics and classification studies of these strains.
